# HPV16-E6 Oncoprotein Activates TGF-*β* and Wnt/*β*-Catenin Pathways in the Epithelium-Mesenchymal Transition of Cataracts in a Transgenic Mouse Model

**DOI:** 10.1155/2018/2847873

**Published:** 2018-05-16

**Authors:** Genaro Rodríguez-Uribe, Nicolas Serafín-Higuera, Gabriela Damian-Morales, Enoc Mariano Cortés-Malagón, Vicky García-Hernández, Odette Verdejo-Torres, Jessica Paulina Campos-Blázquez, Cynthia R. Trejo-Muñoz, Rubén Gerardo Contreras, Rodolfo Ocadiz-Delgado, Carmen Palacios-Reyes, Paul F. Lambert, Anne E. Griep, Teresa Mancilla-Percino, Jaime Escobar-Herrera, Elizabeth Álvarez-Ríos, Carlos Ugarte-Briones, José Moreno, Patricio Gariglio, José Bonilla-Delgado

**Affiliations:** ^1^Department of Genetics and Molecular Biology, Centro de Investigación y de Estudios Avanzados del Instituto Politécnico Nacional (CINVESTAV-IPN), Ciudad de México, Mexico; ^2^Unit of Health Sciences, Faculty of Odontology, Universidad Autónoma de Baja California, Mexicali, BC, Mexico; ^3^Research Unit, Laboratory of Genetics and Molecular Diagnosis, Hospital Juárez de México, Ciudad de México, Mexico; ^4^Department of Physiology Biophysics and Neurosciences, Centro de Investigación y de Estudios Avanzados del Instituto Politécnico Nacional (CINVESTAV-IPN), Ciudad de México, Mexico; ^5^Escuela Superior de Medicina, Instituto Politécnico Nacional, Ciudad de México, Mexico; ^6^McArdle Laboratory for Cancer Research, University of Wisconsin, School of Medicine and Public Health, Madison, Wisconsin, USA; ^7^Department of Cell and Regenerative Biology, School of Medicine and Public Health, University of Wisconsin, Madison, WI, USA; ^8^Department of Chemistry, Centro de Investigación y de Estudios Avanzados del Instituto Politécnico Nacional (CINVESTAV-IPN), Ciudad de México, Mexico; ^9^Department of Cellular Biology, Centro de Investigación y de Estudios Avanzados del Instituto Politécnico Nacional (CINVESTAV-IPN), Ciudad de México, Mexico

## Abstract

**Objective:**

This work aimed to determine if cataractous changes associated with EMT occurring in the K14E6 mice lenses are associated with TGF-*β* and Wnt/*β*-catenin signaling activation.

**Materials and Methods:**

Cataracts of K14E6 mice were analysed histologically; and components of TGF-*β* and Wnt/*β*-catenin signaling were evaluated by Western blot, RT-qPCR, in situ RT-PCR, IHC, or IF technics. Metalloproteinases involved in EMT were also assayed using zymography. The endogenous stabilisation of Smad7 protein was also assessed using an HDAC inhibitor.

**Results:**

The K14E6 mice, which displayed binocular cataracts in 100% of the animals, exhibited loss of tissue organisation, cortical liquefaction, and an increase in the number of hyperproliferative-nucleated cells with mesenchymal-like characteristics in the lenses. Changes in lenses' cell morphology were due to actin filaments reorganisation, activation of TGF-*β* and Wnt/*β*-catenin pathways, and the accumulation of MTA1 protein. Finally, the stabilisation of Smad7 protein diminishes cell proliferation, as well as MTA1 protein levels.

**Conclusion:**

The HPV16-E6 oncoprotein induces EMT in transgenic mice cataracts. The molecular mechanism may involve TGF-*β* and Wnt/*β*-catenin pathways, suggesting that the K14E6 transgenic mouse could be a useful model for the study or treatment of EMT-induced cataracts.

## 1. Introduction

The term cataract refers to the opacification of the crystalline lens, and it is the most common cause of visual loss in humans [1]. Although surgery is the most effective treatment to remove cataracts, a common postoperative complication consists in the development of a secondary cataract, known as posterior capsule opacification (PCO), caused by a fibrotic growth of residual lens epithelial cells left behind in the capsular bag after surgery [2].

It is reported that TGF-*β* pathway stimulates lens epithelial cells to undergo aberrant morphologic and molecular changes that mimic those documented in some forms of human PCO [3]; for example, it was found that intravitreal injection of human recombinant TGF-*β*2 induces cataracts in rat [4]. Furthermore, lens epithelial explants cultured in the presence of TGF-*β*1 developed cataractous changes characterised by an accumulation of fibrous/collagenous extracellular matrix (ECM), along with changes in the lens' cell morphology, which resembles a “mesenchymal-like” phenotype, a process known as “epithelial-mesenchymal” transition (EMT) [5]. During EMT, the epithelial cell changes its morphology and transcriptional landscape to those resembling mesenchymal cells. During cataract formation, lens epithelium-derived myofibroblasts become capable of expressing components of the fibrous ECM, as well as matrix-degrading enzymes [3].

After TGF-*β* receptors activation, the assembly of a receptor complex phosphorylates intracellular proteins of the Smad family, inducing the formation of the p-Smad2/3-Smad4 complex that enters into the nucleus to regulate the transcription of target genes [6]. With particular importance for EMT, TGF-*β* has been shown to activate mitogen-activated protein kinase (MAPK), phosphatidylinositol 3-kinase (PI3K), Rho GTPases, and the Wnt/*β*-catenin pathway; the latter is of crucial importance for EMT of normal and cancerous cells [7–9]. In the absence of Wnt ligands, *β*-catenin forms a “degradation complex” with kinases and scaffold proteins, which phosphorylates *β*-catenin at serine and threonine residues inducing its subsequent ubiquitination by *β*-TrCP for proteasomal degradation [10, 11]. The activation of canonical Wnt signaling induces the phosphorylation of dishevelled protein, allowing its interaction with Axin2 and avoiding the formation of the *β*-catenin degradation complex. The accumulation, and nuclear translocation of *β*-catenin, eventually leads to the activation of Wnt-responsive genes, which regulates cell polarity, proliferation, and differentiation [10, 11].

TGF-*β*1 also stimulates the transcription of the human metastasis-associated protein 1 (MTA1) [12]. MTA1 is a member of the nucleosome remodelling and deacetylation complex (NuRD) with a determinant deacetylase activity for TGF-*β*1-induced EMT phenotype [12]; its expression is also involved in E-cadherin downregulation and supports several cellular functions relevant to the metastatic phenotype including survival, anchorage-independence, migration, and invasion [13].

The K14E6 transgenic mouse expresses the HPV16-E6 oncoprotein under the control of human keratin 14 promoter (K14) [14] and displays cataracts in the lens epithelium in a p53-independent way [15]. The lens consists of a monolayer of cuboidal epithelial cells which covers the anterior surface of a mass of terminally differentiated fibre cells. Fibre cell differentiation initiates in the transitional zone (TZ), where epithelial cells withdraw from the cell cycle, elongate, migrate into the centre of the lens, and synthesise large amounts of crystallin proteins [16, 17]. The transgenic lenses showed by postnatal day 10 an increased number of nucleated cells in the TZ and posterior regions of the lens due to a failure to withdraw from the cell cycle, which finally become multilayered and vacuolated, suggesting a disruption in cell adhesion. Expression of E6 also led to apoptosis, primarily in the fibre cell compartment [15].

This work aimed to determine whether cataractous changes occurring in K14E6 mice lens are associated with EMT. In support of this possibility, we observed many cells with mesenchymal-like morphology and the activation of the TGF-*β* and Wnt/*β*-catenin pathways in K14E6 mouse cataracts. Additionally, target genes involved in EMT were also upregulated, and proteins involved in ECM remodelling such as MMPs were active. Finally, the endogenous stabilisation of Smad7 through an HDAC inhibitor also supports the notion that TGF-*β* pathway could be relevant in EMT in the cataracts of K14E6 transgenic mice. This indicates that K14E6 transgenic mice could be a useful model for the study of EMT and PCO.

## 2. Materials and Methods

### 2.1. Generation of K14E6 Transgenic Mice

The generation and characterisation of K14E6 transgenic mice that express the HPV16-E6 oncogene under the control of the human keratin 14 promoter have been well described previously [14, 18]. The transgene vector contains the HPV16 sequences that include the E6 and E7 ORFs (nucleotides 79–883) with a translation termination linker (TTL) inserted into the E7 ORF, thus causing premature translation termination of E7 sequences. K14E6 transgenic mice were maintained in the FVB/N inbred strain as transgenic homozygotes; they were housed in a pathogen-free barrier facility, and all of the experiments and procedures were approved by the Research Unit for Laboratory Animal Care Committee (UPEAL-CINVESTAV, Mexico; NOM-062-ZOO-1999). For this study, we used 2-month-old male mice from FVB/N nontransgenic (NT) and K14HPV16E6 transgenic (K14E6) strains. All experiments were performed as triplicates and included at least nine mice of each experimental group.

### 2.2. Ocular Tissue Procurement and Histopathological Analysis

The entire ocular organs were dissected, placed in an embedding cassette (Fisher Scientific®), and fixed in 4% paraformaldehyde overnight at 4°C. Eyes were washed in 1x PBS and dehydrated through graded alcohols and xylene. Finally, eyes were paraffin-embedded oriented for 5 *μ*m longitudinal sectioning. The entire eyes were serially sectioned for hematoxylin-eosin staining, in situ RT-PCR, immunohistochemistry, or immunofluorescence procedures. Dissected lenses were obtained for total RNA or protein purification for subsequent RT-PCR or Western blot techniques. No defects or histological alterations were observed in dissected lenses from NT animals.

### 2.3. Genotyping and mRNA Expression of the E6 Transgene

To identify the E6 alleles and their expression, a liquid-phase PCR was performed using E6 specific primers (Table 1). Genomic DNA was isolated as described in the literature [19] and PCR amplification was carried out. The E6 alleles amplification was performed using 40 cycles comprising 30 s denaturing step at 95°C, 1 min annealing step at 60°C, and 30 s elongation step at 72°C. In all genotyping experiments, the amplification of a known DNA sequence (p21/WAF1 gene) was included to ensure the integrity of genomic DNA samples. For the analysis of E6′ mRNA expression, a total RNA was isolated from the lens obtained from transgenic K14E6 and NT mice using the TRIzol protocol (Invitrogen®). Three micrograms of total RNA were reverse-transcribed following the manufacturer's specifications (Invitrogen), and cDNA was amplified through 40 cycles of a 3-step PCR, including 10 s of denaturation at 95°C, 10 s primer-dependent annealing phase (60°C), and 10 s template-dependent elongation at 72°C. GAPDH mRNA was used as endogenous control.

### 2.4. Real-Time Quantitative PCR (RT-qPCR)

The relative quantification of the investigated mRNAs (COL1A1, FN1, Snail, MMP2, MMP9, Wnt1, Wnt3a, Wnt4, Wnt5a, Wnt7a, and MTA1) by RT-qPCR was performed using a 7300 Real-Time PCR System (Applied Biosystems®) as described previously [20, 21]. Oligonucleotide primers were designed to be intron spanning (Table 1), and sequences were obtained from the GenBank database. Optimal PCR reaction for all investigated genes was established using the Maxima SYBR Green/Rox qPCR Master Mix (2x) (Fermentas®), according to the manufacturer's instructions. PCRs were processed through 40 cycles of a 3-step PCR reaction, including 30 s of denaturation at 94°C, 60 s primer-dependent annealing phase (60°C), and 30 s template-dependent elongation at 72°C. The amplification of each template was performed as triplicates in one PCR run. The data obtained in RT-qPCR assays were analysed using the equation described by Livak and Schmittgen [22] as amount of target = 2^−ΔΔCT^. The differential expression of the investigated genes was calculated as the ratio normalised to GAPDH mRNA.

### 2.5. In Situ RT-PCR

For in situ E-cadherin mRNA amplification, a direct in situ RT-PCR was performed as previously described with [23]. Briefly, dried dewaxed sections on DNase/RNase-free electrocharged slides were incubated with proteinase K. After proteinase K digestion, ocular tissues obtained from NT and K14E6 mice were treated with 1 U/sample of DNase I, RNase-free (Roche®) during 48 h at room temperature (2 × 24 h). After thoroughly washing with DEPC-treated water, reverse transcription was performed using the SuperScript II reverse transcriptase (Invitrogen), following the manufacturer's specifications. Slides were incubated at 42°C for 1 h in a sealed humidified chamber. After thoroughly washing with DEPC-treated water, PCR optimal solution (master mix) containing digoxigenin-11-(2′-deoxy-uridine-5′)-triphosphate (DIG-11-dUTP; Roche) was added. Negative controls were made without primers or Taq DNA polymerase. In situ PCR amplification (18 cycles) was performed using the system provided by Perkin Elmer®. Negative controls for in situ RT-PCR were the substitution of one of the primers by H_2_O in the PCR reaction, or the reverse transcription reaction was omitted (not shown). Detection of in situ PCR products was carried out by an indirect immunolabeling method using a primary anti-digoxigenin antibody (Fab fragments; Roche) conjugated to alkaline phosphatase (Sigma®) for 2 h at room temperature. Detection of alkaline phosphatase was performed for 10–30 min using NBT/BCIP kit (Zymed®). After detection, slides were rinsed in distilled water for 5 min, counterstained for 7 min with Fast Green (Sigma), washed, dehydrated, and air-dried before mounting in Permount histological mounting medium (Fisher Scientific®).

### 2.6. Immunohistochemistry

Tissue sections obtained from paraffin-embedded eyes from 2-month-old mice were deparaffinized and rehydrated as described previously [24]. Protein detection was performed using the Mouse-Rabbit PolyDetector HRP/DAB Detection System (Bio SB®) according to manufacturer's specifications. Tissue sections were incubated for 12–16 h at 4°C with a primary antibody against the E6 oncoprotein, PCNA, TGF-*β* RI, TGF-*β* RII, TGF-*β*1, TGF-*β*2, MTA1, vimentin (Santa Cruz Biotechnology®, ct. numbers: SC-1583, SC-25280, SC-101574, SC-17791, SC-130348, SC-374658, SC-373765, and SC-66002, resp.), p-Ser465/467-Smad 2, active-*β*-catenin, or Smad7 (Millipore®, cat. numbers: AB3849, 05-665, and ST1625, resp.), following manufacturer's recommended protocol. Following three washes in 1x PBS, sections were incubated with PolyDetector DAB chromogen (0.3 mg/ml DAB; Bio SB®), counterstained with hematoxylin, and mounted in Permount reagent (Fisher Scientific). Tissues without primary antibody were included as negative staining controls (not shown).

### 2.7. Immunofluorescence

The immunofluorescence detection of E-cadherin, total *β*-catenin, and Phalloidin (Invitrogen, cat. numbers: 71-1400, 71-7800, and A12381, resp.) was performed on paraffin-embedded eye sections as previously described in detail [25]. As secondary antibodies, we employed antibodies coupled to Alexa Fluor 594 or coupled to Alexa Fluor 488 (Invitrogen). Nuclei were stained in blue with TO-PRO-3 iodide (Invitrogen). Assays omitting primary antibody were included as negative controls (not shown).

### 2.8. Western Blotting

Lenses were lysed with RIPA buffer, and total protein extracts were denatured for 5 minutes at 100°C. 30 *μ*g of total proteins lysates was resolved in SDS-PAGE and transferred to nitrocellulose or PVDF membranes as indicated previously [26]. Membranes were blocked for 1 h in 1x PBS with 5% nonfat dry milk and then incubated with primary antibody diluted at 1 : 500, washed three times, and incubated with appropriated horseradish peroxidase-conjugated secondary antibody and the antigen-antibody reaction was detected using SuperSignal West Pico Chemiluminescent Substrate (Thermo Scientific) according to the manufacturer's recommendations.

### 2.9. Zymography

MMP9 and MMP2 activity in mice was measured by gelatin zymography. The crystalline lysates were mixed with sample buffer in 3 : 1 proportion (2.5% SDS, 1% sucrose, and 4 g/ml phenol red) without reducing agent and loaded to 8% acrylamide gels copolymerized with gelatin at 1 mg/ml. Gels were rinsed three times in 2.5% Triton X-100 and then incubated in assay buffer (50 mM Tris-HCl pH 7.4, 5 mM CaCl_2_) at 37°C for 48 h. Gels were fixed and stained with 0.25% Coomassie Brilliant Blue G-250 in 10% acetic acid and 30% methanol. Proteolytic activity was detected as clear bands against the background stain of an undigested substrate. Densitometry was performed using Fiji software (ImageJ ver. 2.0).

### 2.10. Molecular Docking Assay

A series of fourteen 2-substituted isoindolines derived from *α*-amino acids were synthesised and patented by the group of Mantilla et al. (Patent Registration Number: Mx/a/2014/002124) [27]. These compounds were analysed cursive by molecular docking to evaluate whether these compounds could act as HDACs inhibitors. Briefly, structural formulas were drawn with the Isis/Draw software and converted into their three-dimensional format using the WebLab Viewer and Molekel Visualization Package. First, an optimisation of the isoindoline geometry (energy minimisation) was carried out with the Gaussian 98 version A.3 software. Second, on the 3D structure of the* Homo sapiens *HDAC8 protein (PDB: 1T64) using the B3LYP 6-31G algorithm, the molecular docking was carried out with a version of AutoDock 4.2, whose algorithm allows the complete flexibility of small ligands. The preparation of ligand structures and proteins, as well as the definition of binding sites, was carried out under a GRID base. The coupling of HDACs and isoindolines was performed with the hybrid genetic Lamarckian algorithm. Parameters for molecular docking were the following: population: 100, evaluations: 10,000,000. All proteins visualisations were carried out through the “Visual Molecular Dynamics” software.

### 2.11. Isoindoline Selection and Treatment Protocol

Based on the molecular docking, we selected the isoindoline derivative from L-leucine (2b) since it exhibited more negative Δ*G* values for the HDAC1-2b complex in the catalytic site. K14E6 mice were injected intraperitoneally with isoindoline 2b at two dosages, 10 *μ*M/Kg and 100 *μ*M/Kg, every 12 h during three days. After treatment, ocular organs were resected for histological evaluations such as H&E staining and immunohistochemistry of Smad7, PCNA, and MTA1 proteins. Negative control was considered as K14E6 mice treated with vehicle (injectable water) and as the positive control, the group treated with TSA (dose: 0.01 *μ*M/Kg). All compounds were well tolerated at the indicated dosages, and no toxic effects were reported during isoindoline 2b treatment. Importantly, same dosages for TSA and isoindoline 2b were unable to be compared due to TSA toxicity [28].

### 2.12. Digital Image Analysis and Quantification

All images were digitally analysed using the Image-Pro Plus Analysis Software (version 4.5.0.19, Media Cybernetics®) as described elsewhere [20]. GAPDH signal was used to normalise data in case of in situ RT-PCR quantification.

### 2.13. Statistical Analysis

Data are presented as the mean ± standard deviation (SD). Statistical evaluation of significant differences was performed using Student's *t*-test. Differences of (*∗*) *p* < 0.05, (*∗∗*) *p* < 0.01, and (*∗∗∗*) *p* < 0.001 were considered statistically significant.

## 3. Results

### 3.1. Validation of the Transgenic Mouse Model in the Lens

**Figure 1:**
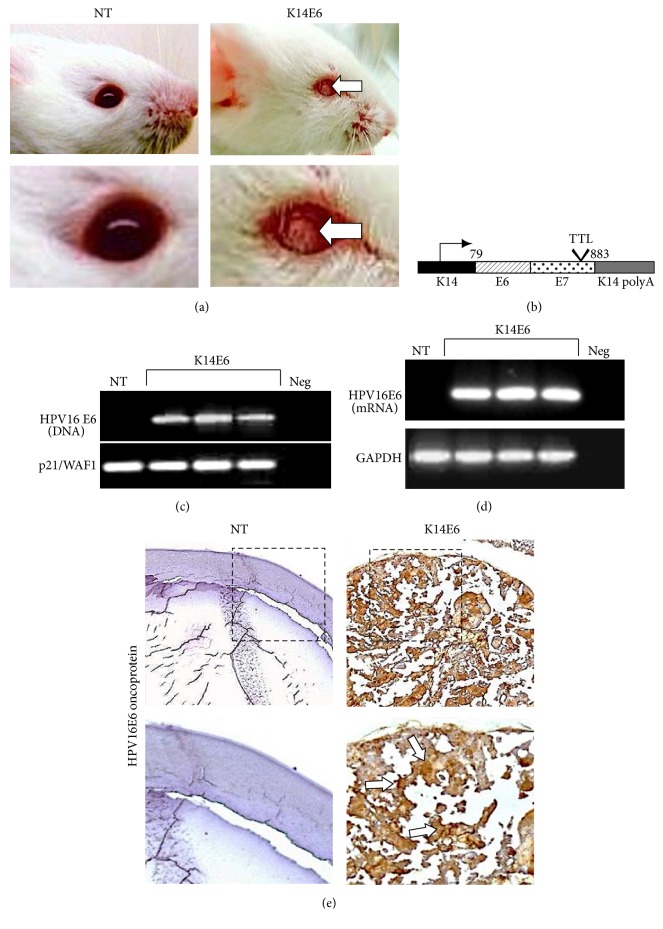
*Transgenic mice expressing the human papillomavirus E6 oncogene develop cataracts*. (a) Representative images of NT and K14E6 transgenic eyes. 2-month-old K14E6 mice expressing the HPV16-E6 oncoprotein develop cataracts (white arrows) in comparison with NT mice. (b) Genetic map of the K14E6 transgene construction. Mice expressing the E6 oncogene contain a DNA sequence that overlaps the HPV16-E6 and E7 ORFs spanning nucleotides 79–883 under the control of the human keratin 14 promoter (K14). Transgenic mice have a translational termination linker (TTL) in the 3′ E7 gene precluding E7 translation. (c) Presence of the viral E6 oncogene in K14E6 transgenic mice. Amplification control of p21/WAF1 DNA sequence in NT and K14E6 mice was used to ensure the integrity of genomic DNA samples. Negative control (Neg): PCR reaction without DNA. (d) Endpoint RT-PCR determined the expression of the HPV16-E6 transcript in the mouse lens. Expression control (GAPDH mRNA). Negative control (Neg): PCR reaction without cDNA. (e) Expression of the HPV16-E6 oncoprotein by immunohistochemistry. White arrows indicate zones of high E6 expression. Magnifications: (e) (10x, inset: 20x).

We observed cataract formation in the lens of all 2-month-old K14E6 transgenic mice (Figure 1(a)). The K14E6 mouse contains the HPV16-E6 oncogene flanked by the human cytokeratin 14 (K14) promoter, the K14 polyadenylation sequences (K14 polyA), and a translational termination linker (TTL) inserted into the 3′ region of the E7 oncogene, so that translation of E7 is disrupted (Figure 1(b)). The HPV16-E6 transgene was detected by PCR employing the DNA from the transgenic mice tail (Figure 1(c)). To verify the expression of E6 oncogene, the lenses of 2-month-old nontransgenic (NT) and transgenic mice (K14E6) were dissected, and total RNA was extracted. As expected, the E6 mRNA expression was observed in the lens of transgenic mice (Figure 1(d)). Furthermore, immunohistochemistry determination was carried out to detect the E6 oncoprotein. We found a positive signal only in K14E6 mice lens (Figure 1(e)).

### 3.2. Histological Changes in Transgenic Mice Lens

**Figure 2:**
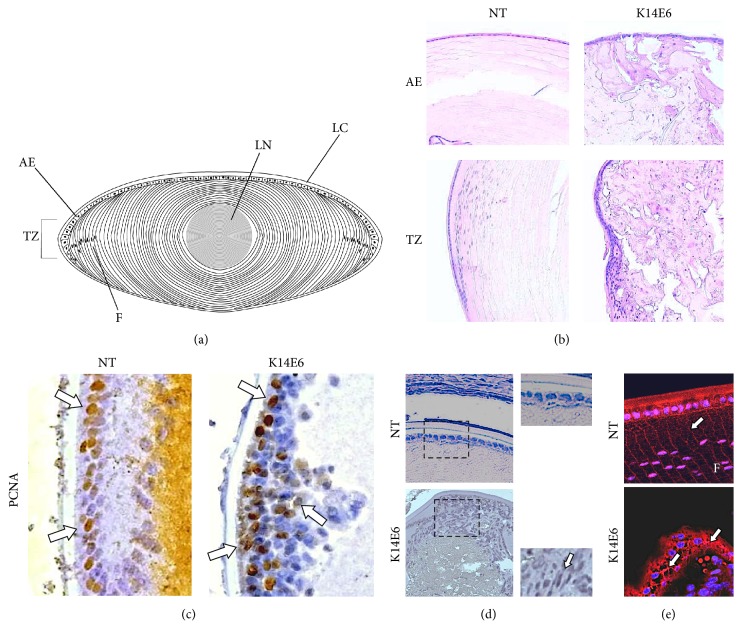
*Cataracts show abnormal localisation of nucleated and proliferating cells in K14E6 transgenic mice*. (a) Schematic diagram of the mammalian lens. LC: lens capsule, AE: anterior epithelium, TZ: transitional zone, F: lens fibres, and LN: lens nucleus. (b) Hematoxylin-Eosin staining of 2-month-old NT and K14E6 transgenic mice with lens showing the AE and TZ tissue organisation. The K14E6 mice show wholly disrupted AE, along with cortical liquefaction and reabsorption. (c) Immunohistochemical detection of PCNA as a cell proliferation marker. The nuclear signal of PCNA ((c) white arrows) was found mainly in the basal layer cells, and it was limited to anterior epithelium in NT, whereas K14E6 exhibited PCNA staining in multiple layers in AE. (d) AE cell morphology shows a simple cuboidal epithelium in NT, whereas the K14E6 transgenic mice exhibited cells with mesenchymal-like phenotype ((d) white arrow). (e) Phalloidin staining (red signal, white arrows) shows stress fibres located at cell-cell contacts in differentiated cells in NT mice, whereas K14E6 anterior epithelium shows a disorganised staining pattern. Magnifications: (b) (10x), (c) (40x), (d) (10x, inset: 40x), and (e) (40x).

The architecture of a normal lens is formed by an epithelial cells layer that covers the anterior surface (Figure 2(a)). At the transitional zone (TZ), the epithelial cells differentiate into fibre cells which account for the bulk of the lens. The fibre cells change their expression pattern and lose intracellular organelles during differentiation [16]. These structures can be observed in the lens of NT mice (Figure 2(b)); however, examination of eye sections from 2-month-old K14E6 transgenic mice showed loss of tissue organisation, increase in the number of nucleated cells, cortical liquefaction, and cortical reabsorption (Figure 2(b)). Hyperproliferation in cataracts was assessed by detecting the cell proliferation marker PCNA in the lens by immunohistochemistry. As compared to NT mice, lens sections of K14E6 transgenic mice showed an increase in PCNA-positive cells, suggesting an increased cell proliferation (Figure 2(c)). Further histological examination of cataracts showed cells with mesenchymal-like characteristics (mesenchymal spindle-shaped and fusiform features) (Figure 2(d)). Changes in cell morphology could be due to a reorganisation of filamentous actin. Thus, we performed a phalloidin staining to detect actin-stress fibres in the lens cells of K14E6 mice. As can be seen in Figure 2(e), stress fibres in NT anterior epithelial cell are localised at cell-cell contacts in differentiated cells, whereas K14E6 epithelial cells show a disorganised pattern in the whole anterior epithelium (Figure 2(e)).

### 3.3. TGF-*β* Signaling Pathway in Transgenic Mice Cataract

**Figure 3:**
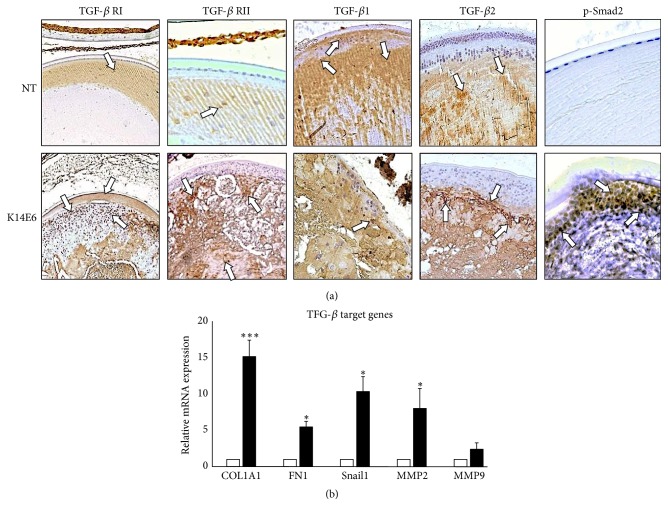
*TGF-β signaling pathway is activated in the lens of K14E6 transgenic mice*. (a) Immunohistochemical detection of TGF-*β* receptors, TGF-*β* ligands, and p-Smad2 in the lens of 2-month-old NT and K14E6 transgenic mice. Arrows indicate the positive signal for each antigen. (b) The expression levels of several TGF-*β* target gene transcripts were assessed by RT-qPCR. Corresponding mRNA levels in the NT mice were given a value of 1.0. The experiments were performed as triplicates, and nine mice of each experimental group were used in each replicate. COL1A1: collagen 1A1, FN1: fibronectin, MMP2: metalloproteinase 2, and MMP9: metalloproteinase 9. Statistically significant values were calculated as described in Materials and Methods. Statistical significance: ^*∗*^*p* < 0.05 and ^*∗∗∗*^*p* < 0.001. Magnification: (a) (10x).

It is well known that TGF-*β* is involved in cataracts' aetiology and it is considered as one of the most potent and better-studied inducers of EMT [29]. Thus, it was important to analyse the expression of TGF-*β* pathway components in cataracts of K14E6 transgenic mice. No significant differences in the expression of TGF-*β* RI, TGF-*β*1, and TGF-*β*2 were detected in the lens of 2-month-old K14E6 transgenic mice as compared to NT mice (Figure 3(a)). However, a high level of TGF-*β* RII and p-Smad2 was detected specifically in the transgenic lenses, indicating an active TGF-*β* signaling (Figure 3(a)). Furthermore, the analysis of TGF-*β*-responsive genes by RT-qPCR showed increased expression of collagen (COL1A1), fibronectin (FN1), Snail, matrix metalloproteinase 2 (MMP2), and matrix metalloproteinase 9 (MMP9) (Figure 3(b)) in the cataracts of transgenic mice as compared to nontransgenic lens samples. Therefore, these results suggest that the TGF-*β* signaling pathway is activated in the cataracts of K14E6 mice.

### 3.4. Expression of EMT Markers in the K14E6 Transgenic Mice Cataracts

**Figure 4:**
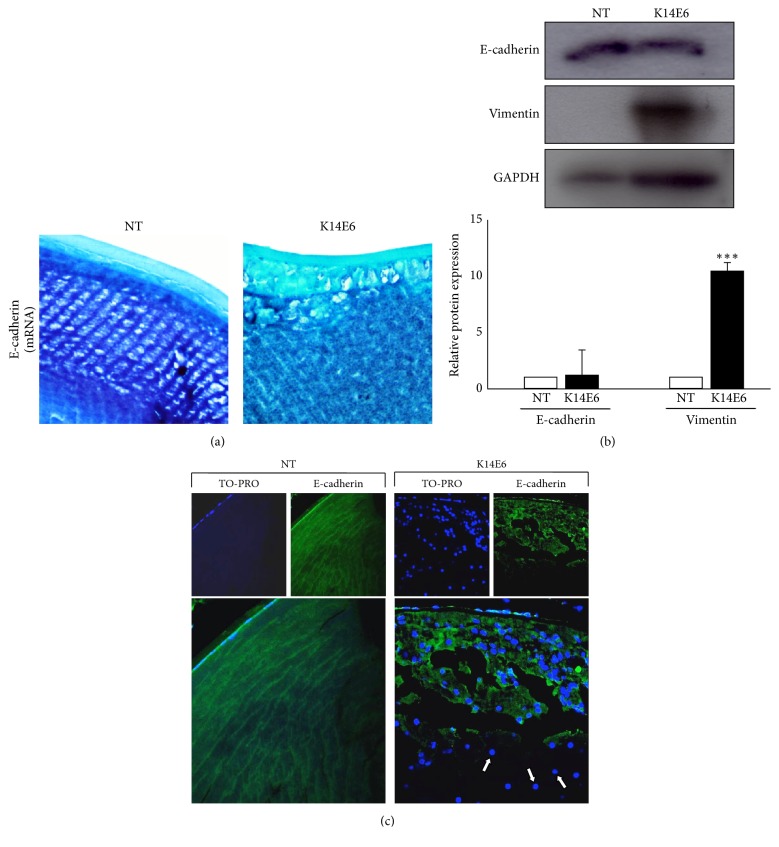
*The E6 oncoprotein induces the expression of EMT markers*. (a) Representative images for E-cadherin mRNA detection by in situ RT-PCR in 2-month-old NT and K14E6 lenses (see Materials and Methods). (b) E-cadherin and vimentin protein expression normalised with GAPDH by Western blot. (c) Immunofluorescence detection of E-cadherin in 2-month-old NT and K14E6 transgenic lens. Green signal: E-cadherin. Blue signal: TO-PRO (cell nuclei). White arrows show nucleated cells that lack E-cadherin signal near the lens nucleus. Statistical significance: ^*∗∗∗*^*p* < 0.001. Magnifications: (c) (40x).

Since downregulation of E-cadherin and upregulation of Vimentin are considered as a hallmark of EMT [29, 30], we studied the expression of these proteins in the lens of 2-month-old NT and K14E6 transgenic mice. Although we detected a slight reduction in E-cadherin mRNA by in situ RT-PCR (Figure 4(a)), total protein levels seem not to be affected (Figure 4(b)). However, the E-cadherin cellular localisation differs notably between mice strains; E-cadherin is localised at the cell membrane in epithelial and fibre cell in NT mice, whereas K14E6 mice show a cytoplasmic distribution and a lack of E-cadherin expression in regions located near the lens' nucleus (see K14E6 strain, Figure 4(c), white arrows). Furthermore, vimentin, a protein highly expressed in EMT, is overexpressed in transgenic mice cataracts (Figure 4(b)). This evidence may suggest that EMT occurs in the cataracts of the K14E6 transgenic mice.

### 3.5. Increased Expression and Activity of MMPs in Cataracts of Transgenic Mice

**Figure 5:**
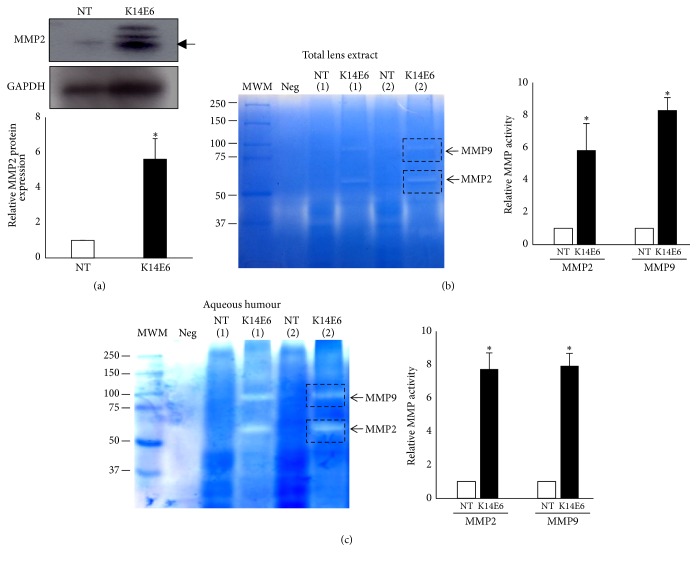
*E6 oncoprotein increases metalloproteinase production and activity*. (a) Western blot analyses of MMP2 in total lens extract of NT and K14E6 mice. (b) Zymogram analyses of MMP9 and MMP2 proteins in total lens extract or aqueous humour (c) in the lens of NT and K14E6 mice. Gelatin degradation was monitored by sodium dodecyl sulphate (SDS) 8% polyacrylamide gel electrophoresis (PAGE) and Coomassie blue staining as described in Materials and Methods. Statistical significance: ^*∗*^*p* < 0.05. MWM: molecular weight marker; Neg: negative control (no sample was included). The experiments were performed as triplicates, and a pool of nine mice of each strain was considered as a biological replica in the zymogram assay.

Since the expressions of matrix metalloproteinases (MMPs) are part of the EMT process [31], we performed a Western blot to determine the protein levels of MMP2, since we previously showed that MMP2 mRNA expression was increased in transgenic mice cataracts (Figure 3(b)). As Figure 5(a) shows, MMP2 augmented its protein expression levels in total lens extracts evaluated by Western blot. We next wondered whether these increased levels of MMP2 are proteolytically active. To assess this concern, we performed zymography assays (Figures 5(b) and 5(c)) to evaluate MMP activity in total lens extracts, as well as in aqueous humour [32]. Our results show an increased activity not only of MMP2 but also of MMP9 in samples of transgenic mice as compared to NT (Figures 5(b) and 5(c)).

### 3.6. Increased Levels of Nuclear *β*-Catenin Protein in the K14E6 Transgenic Mice Cataracts

**Figure 6:**
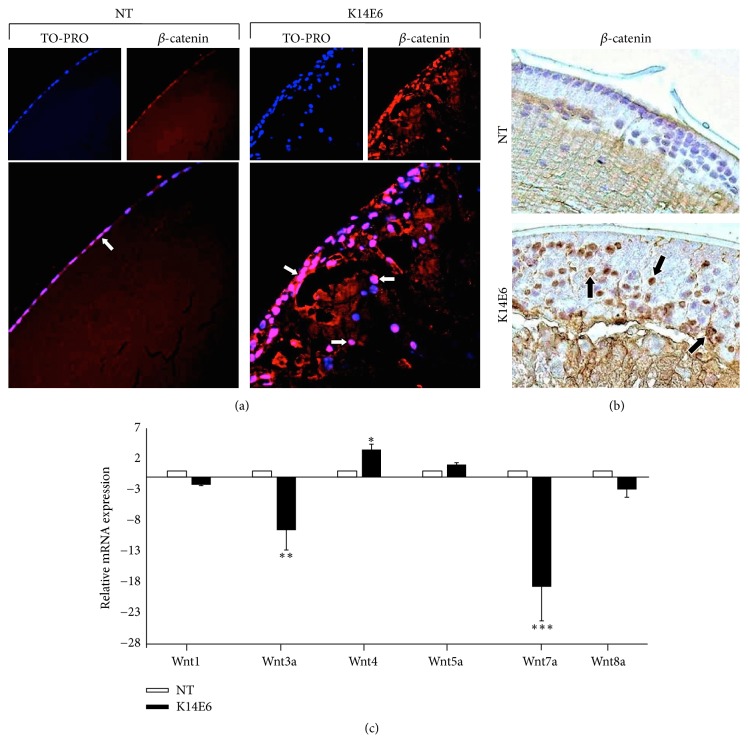
*Nuclear β-catenin increases in the lens cells of K14E6 transgenic mice*. (a) Immunofluorescent detection of *β*-catenin (red signal) in the lens of 2-month-old NT and K14E6 transgenic mice. Merged signal (purple signal) shows nuclear *β*-catenin in the anterior epithelium (white arrows). (b) Immunohistochemical detection of *β*-catenin protein is indicated as a brown nuclear signal (black arrows). (c) mRNA expression of selected Wnt ligands assessed by RT-qPCR. Wnt4 ligand was the most overexpressed, and Wnt7a was the most subexpressed ligand in the K14E6 lens. The experiments were performed as triplicates (3 mice peer biological replica). Statistical significance: ^*∗*^*p* < 0.05, ^*∗∗*^*p* < 0.01, and ^*∗∗∗*^*p* < 0.001. Magnification: (a) and (b) (40x).

A previous report indicates that Wnt signaling is involved in TGF-*β*-induced EMT during the development of fibrotic plaques in the lens [7]. Since nuclear translocation of *β*-catenin is a requisite for Wnt pathway activation [33], we analysed the cellular localisation of *β*-catenin in the cataracts of K14E6 mice by immunofluorescence (Figure 6(a)) or immunohistochemistry (Figure 6(b)). As Figures 6(a) and 6(b) indicate, *β*-catenin protein exhibits cytoplasmic and nuclear localisation in the K14E6 mice. Additionally, the mRNA of Wnt ligands was assayed by RT-qPCR, and Wnt4 and Wnt5a ligands were upregulated in K14E6 cataracts, whereas Wnt1, 3a, 7a, and 8a, were downregulated. Interestingly, Wnt7a was the Wnt ligand that had the most profound differential expression in comparison to NT mice (Figure 6(c)) (see Discussion). Together, the results suggest a possible induction of the Wnt signaling pathway in the K14E6 mice cataracts.

### 3.7. MTA1 Induction in K14E6 Cataracts

**Figure 7:**
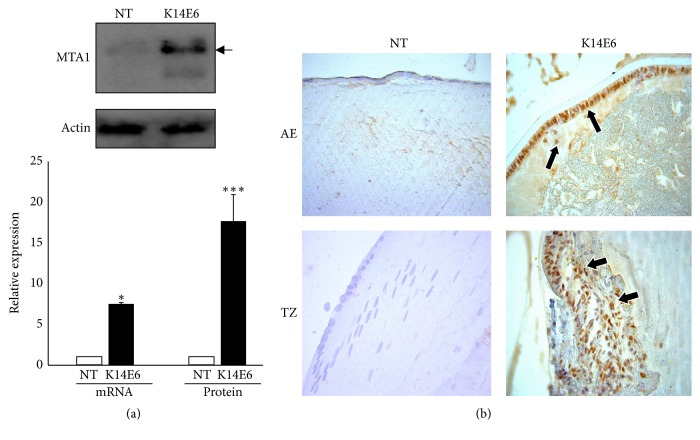
*The MTA1 nuclear receptor increases its expression in K14E6 mice lens*. (a) mRNA and protein expression of MTA1 evaluated by RT-qPCR or Western blot, respectively. (b) Immunohistochemistry detection of MTA1 protein in the lens of NT and K14E6 transgenic mice. As observed, the MTA1-positive nuclear signal in AE and TZ was higher in K14E6 transgenic mice lenses (Black arrows), as compared to NT lenses. Statistical significance: ^*∗*^*p* < 0.05 and ^*∗∗∗*^*p* < 0.001. Magnification: (b) (40x).

MTA protein family members form independent NuRD complexes capable of recruiting HDAC activity onto target genes [34]. In particular, metastasis-associated protein 1 (MTA1) has been identified as an integral member of NuRD complexes [35], and it is associated with EMT during invasiveness of several types of cancers [12, 36]. As MTA1 gene is upregulated by TGF-*β* signaling [12], we next wondered whether the mesenchymal-like phenotype observed in the lens' epithelial cells is due to an enhanced expression of MTA1 gene. As Figure 7 shows, MTA1 mRNA (Figure 7(a)) and protein (Figures 7(a) and 7(b)) increase their expression levels in the anterior epithelial cells and transitional zone in K16E6 mice cataracts.

### 3.8. Selection of a 2-Substituted Isoindoline as HDAC Inhibitor

HDACs have an important role in EMT and cancer. Therefore, several compounds have been synthesised as HDAC inhibitors. Classical groups of HDAC inhibitors (iHDACs) include short chains of fatty acids (such as valproic acid), hydroxamic acids (such as trichostatin A or TSA), cyclic tetrapeptides, or benzamides [37].

**Figure 8:**
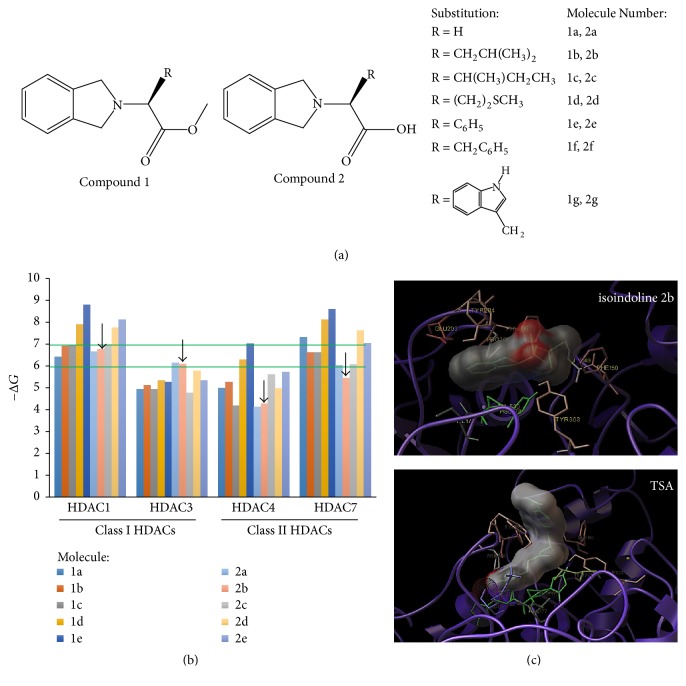
*Docking evaluation of isoindolines 2-substituted in HDAC catalytic sites*. (a) Structure of 14 isoindolines 2-substituted with an ester group (Compound 1) or with a carboxyl group (Compound 2). (b) Δ*G* interaction values (Kcal/mol) of 10 2-substituted isoindolines that showed affinity for the catalytic site of HDAC1, 3, 4, and 7. Green horizontal lines indicate the binding selectivity of isoindoline 2b for the catalytic site of HDAC1 versus HDAC7. (c) Schematic representation of noncovalent interactions of isoindoline 2b versus TSA for the HDAC1 catalytic site.

In recent years, the group of Mantilla et al. synthesised 14 2-substituted isoindolines derived from *α*-amino acids (Patent Registration Number: Mx/a/2014/002124) [27] (see Figure 8(a)). Each 2-substituted isoindoline was derived from an isoindoline ring with an ester group (compound 1) or a carboxyl group (Compound 2). Next, we tested the binding of these 14 compounds into HDACs catalytic sites. As Figure 8(b) shows, docking assays indicate that 10 of 14 compounds display a spontaneous affinity for the active site of HDACs 1 and 3 (class I HDACs) and HDACs 4 and 7 (class II HDACs).

As the Δ*G* values indicate, the 10 selected isoindolines showed a preferential affinity for the catalytic sites of HDACs 1 and 7 (see Figure 8(b)), as compared to HDACs 3 and 4. Since HDACs also participate in normal cell homeostasis [38], we were interested in this in silico selectivity for HDAC1 and 7, since HDAC pan-inhibitors, such as TSA, increase the rate of cytotoxic effects. Because isoindoline 2b is more selective for the HDAC1 enzyme (Δ*G* ≈ −7 Kcal/mol) than HDAC7 (Δ*G* ≈ −6 Kcal/mol) (see green horizontal lines in Figure 8(b)), we choose isoindoline 2b as a selective iHDAC for HDAC1 enzyme.  Figure 8(c) shows a schematic representation of all noncovalent interactions of isoindoline 2b versus TSA in the catalytic site of HDAC1.

### 3.9. Isoindoline 2b Augments Smad7 Protein and Suppresses the Expression of TGF-*β*-Responsive Genes

Smad7 protein, an endogenous inhibitor of TGF-*β* pathway, has been reported to be posttranslationally regulated by HAT/HDAC activity [38–40]. Direct acetylation of Smad7 by p300 on amino acids K64 and K70 promotes its stabilisation [39], whereas deacetylation of these residues by HDAC1 induces its proteasomal degradation [40]. As Docking assays suggested a preferential inhibition for HDAC1 enzyme by isoindoline 2b (Figure 8), we next wondered whether the isoindoline 2b treatment could stabilise Smad7 protein and thus inhibiting TGF-*β* signaling.

**Figure 9:**
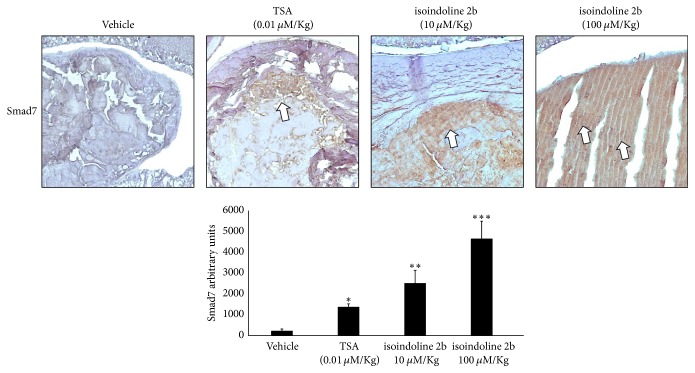
*Smad7 protein increases in a dose-dependent manner by isoindoline 2b iHDAC*. Immunohistochemistry detection of Smad7 protein (showed by white arrows) in the lenses of K14E6 transgenic mice treated with two distinct nontoxic dosages of isoindoline 2b iHDAC versus a nontoxic dosage of TSA. Statistical significance: ^*∗*^*p* < 0.05 and ^*∗∗∗*^*p* < 0.001. Magnification: 10x.

**Figure 10:**
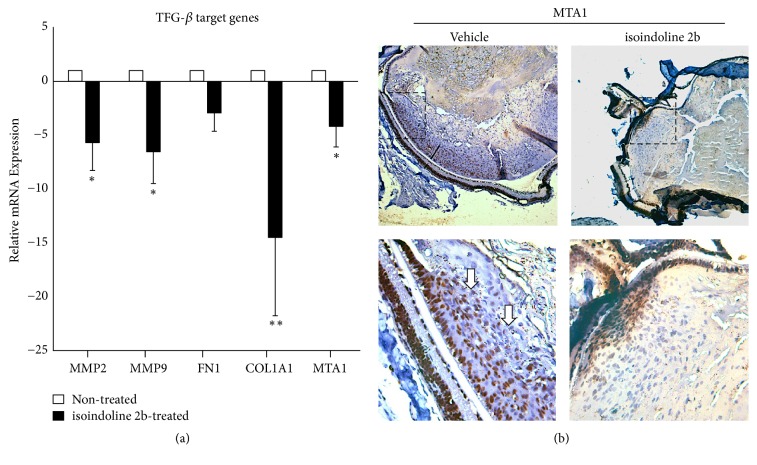
*Isoindoline 2b suppresses TGF-β-responsive genes in K14E6 cataracts*. (a) mRNA detection of TGF-*β*-responsive genes such as MMP2, MMP9, FN1, COL1A1, and MTA1 evaluated by RT-qPCR in the lenses of K14E6 mice (treated versus control). (b) Immunohistochemical detection of MTA1 protein (showed by white arrows) in the lenses of K14E6 transgenic mice (treated versus control). Isoindoline treatment dosage: 100 *μ*M/Kg. Statistical significance: ^*∗*^*p* < 0.05 and ^*∗∗*^*p* < 0.01. Magnification: (b) (10x, inset: 40x).

We evaluated two nontoxic dosages of isoindoline 2b by intraperitoneal injections in K14E6 mice strain (see “Materials and Methods”). As Figure 9 indicates, isoindoline 2b iHDAC was superior to a nontoxic dosage of TSA in increasing Smad7 protein levels in the lenses of K14E6 mice in a dose-dependent manner (Figure 9). Thus, we selected the 100 *μ*M/Kg dosage of isoindoline 2b to evaluate whether the augmented protein levels of Smad7 protein diminish the TGF-*β*-responsive genes overexpressed in K14E6 lenses. As Figure 10(a) indicates, MTA1, MMP2, MMP9, FN1, and COL1A1 mRNAs were downregulated in the K14E6-treated group. In particular, MTA1 mRNA (Figure 10(a)) and protein (Figure 10(b)) also diminish in the K14E6 cataracts.

**Figure 11:**
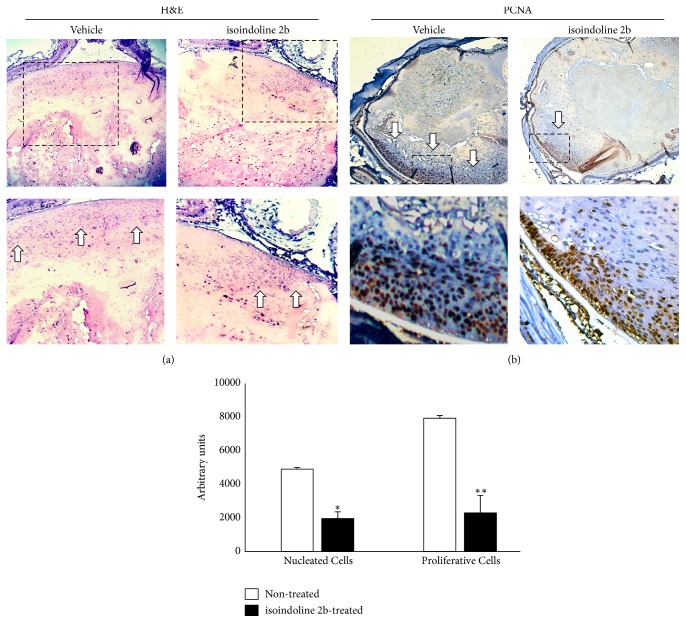
*Isoindoline 2b reduces the number of proliferative-nucleated cells in K14E6 cataracts*. (a) Hematoxylin-Eosin staining of treated versus control K14E6 mice lenses showing a reduction in the nucleated cell population that approaches the lens' nucleus. (b) Immunohistochemical detection of PCNA showing also a reduction in proliferative-immature cells (indicated by white arrows). Isoindoline dosage: 100 *μ*M/Kg. Statistical significance: ^*∗*^*p* < 0.05 and ^*∗∗*^*p* < 0.01. Magnification: (10x, inset: 40x).

Finally, we analysed the histopathology of K14E6 mice after treatment with intraperitoneal dosages of isoindoline 2b (100 *μ*M/Kg dosages). As Figure 11 shows, the number of proliferative-nucleated cells is reduced in the lens' nucleus of treated mice as compared to control, supporting the evidence that the K14E6 mice develop cataractous lesions in a TGF-*β*-dependent manner.

## 4. Discussion

In this study, we propose that the presence of HPV16-E6 oncoprotein induces EMT in the lens cells of transgenic mice, since our results showed cells with mesenchymal-like characteristics along with the activation of signaling pathways related to EMT.

The expression of the HPV16-E6 oncoprotein throughout the lens epithelium in K14E6 mice was already demonstrated in 10-day-old K14E6 transgenic mice by Chong et al. [7]. They reported that the E6 oncoprotein expression in the lens resulted in the alterations of the structural organisation, which include cortical liquefaction and reabsorption. Histological findings reported in this study also show cortical hyperproliferation with an increased number of nucleated cells in the fibre cell compartment in 2-month-old transgenic mice lens. Therefore, we suggested that the E6-derived phenotype in the lens is age-independent.

It is well documented that EMT is an essential process in the development of human cataracts [17] and PCO [2]. In these processes, cells with a fibroblastic morphology migrate onto the posterior lens capsule to deposit large amounts of extracellular matrix (ECM) in clinical and experimental models [41]. Furthermore, it is documented that TGF-*β* induces the reorganisation of actin filaments during cell-ECM interactions in the lens epithelial cell via Rho-GTPase activation [42], playing a crucial role in lens' epithelial cell proliferation, migration, elongation, and survival [43]. We also observed actin reorganisation in the nucleus region of K14E6 transgenic mice lenses (Figure 2). All the above suggest that the EMT process is involved in the development of E6-induced cataracts.

To gain additional insights that EMT is involved in E6-induced cataracts, we analysed pathways implicated in EMT. For example, TGF-*β* pathway activation is a distinctive mark of some forms of human cataracts which displays EMT [44]. Moreover, TGF-*β*2 induces the development of cataract* in vitro* in rat lens [4]. Notably, we observed an increased nuclear signal of p-Smad2 in transgenic mice cataracts, suggesting that the TGF-*β* pathway is active in K14E6 cataracts. Since quiescent fibre cells in NT mice minimally express TGF-*β* receptors and ligands (See Figure 2), p-Smad2 detection is an adequate criterion to evaluate TGF-*β* signaling activation in K14E6 cataracts.

It has been reported that E-cadherin, a membrane-bound epithelial cell marker, and vimentin, a type III intermediate filament mesenchymal cell marker, have been used to describe EMT [45]. Downregulation of E-cadherin in TGF-*β*-induced EMT has been demonstrated in cultured lens epithelial cells [46] and lens epithelial explants [46, 47]. Our observations show a disorganised and reduced expression of E-cadherin protein in some areas of K14E6 lens nucleus (see Figure 4(c)). Several transcriptional repressors of E-cadherin have been identified, including the Snail superfamily of zinc-finger transcription factors [48]. Snail is considered an effector of TGF-*β*-induced EMT and it is activated by TGF-*β* signaling through the action of p-Smad2/3 and Smad4 in mouse lens epithelial cells [49]; consistent with these reports, we also observed increased expression of Snail mRNA in K14E6 cataracts. Additionally, vimentin is a protein induced by Snail [50], and its* in vivo *overexpression in transgenic mice also inhibits the normal fibre cell differentiation, leading to cataract formation [51]. In agreement with this, we observed that vimentin expression was also increased in the cataract of the K14E6 transgenic mice (see Figure 4(b)).

Another characteristic of the mesenchymal cells is the capacity to produce extracellular matrix. This is a dynamic process that implicates the expression of proteins that participate in the formation (fibronectin and collagen) and degradation of cellular structures (MMP2 and MMP9) [52]. The matrix metalloproteinases (MMPs) are a family of zinc-dependent matrix-degrading enzymes involved in multiple diseases including tissue fibrosis, with emerging roles in a variety of cataract phenotypes, particularly in anterior subcapsular cataracts (ASC), and PCO [53–55]. MMP2 is capable of cleaving ECM components such as collagen type IV, exposing sites of attachment that can promote cell migration [56]. Additionally, MMP9 may play a more upstream role in TGF-*β*-induced ASC than MMP2 [31, 57]. Interestingly, our results showed increased expression of MMP2 protein and MMP2/MMP9 activity in total proteins extracts and aqueous humour in 2-month-old K14E6 mice cataracts.

Wnt signaling is another pathway reported in TGF-*β*-induced EMT, which is also activated during fibrotic changes in lens cells [7]. Nuclear localisation of *β*-catenin is evident after exposure of epithelial lens explants to TGF-*β*2 [7], and our data also show increased nuclear *β*-catenin staining in K14E6 cataracts (see Figure 6), which is consistent with previous observations [7]. Furthermore, it was previously reported that Wnt signaling is also induced by the HPV16 E6 oncogene in the skin of K14E6 transgenic mice [58]. We also analysed the Wnt ligands expression in K14E6-induced cataracts, since they stabilise *β*-catenin. We observed increased expression of the mRNA of Wnt4 and Wnt5a as Wnt inducers but not other classical Wnt inducers such as Wnt1, Wnt3a, or Wnt8a. In support of our observations, it is reported that Wnt ligands are also overexpressed in the anterior subcapsular plaques of transgenic mice that overexpress TGF-*β*1 in the lens [7], supporting again the possibility that Wnt signaling is involved in the cataracts formation in K14E6 mice.

One emerging group of chromatin modifiers and coregulators are the members of the metastasis-associated (MTA) protein family. This family comprises three different proteins (MTA1, MTA2, and MTA3), which are integral parts of the NuRD (Nucleosome Remodeling and Histone Deacetylation) complexes that have essential transcriptional regulatory functions via histone deacetylation and chromatin remodelling. The MTA family of proteins was found to have an important role in EMT regulation [36]. In particular, MTA1 induced the EMT in mammary epithelial cells [59] and was also reported to be transcriptionally upregulated by TGF-*β*1 in stimulated mammary epithelial cells [12]. K14E6 mice cataracts also overexpress MTA1 (mRNA and protein), an effect probably regulated in a TGF-*β*-dependent manner (see Figures 3 and 7).

Finally, the Smad7 protein stabilisation (see Figure 9), through a selective* in vivo *inhibition of the HDAC1 catalytic site (see Figure 8), clearly demonstrates that the endogenous inhibition of TGF-*β* signal-responsive genes (Figure 10) may lead to a reduction in the proliferative-nucleated cells located in the lens nucleus of iHDAC-treated K14E6 mice (Figure 11). Smad7 protein is controlled endogenously by direct HAT [39] and HDAC [40] covalent modifications. Acetylation of lysine 64 and 70 by p300 protects Smad7 against the proteasomal degradation via Smurf1 ubiquitin ligase [39]. As Smurf1 ubiquitylates the nonacetylated region comprising lysines 64 and 70, the Smad7 stabilisation depends on a competition between acetylation and deacetylation [38]. As HDAC1 deacetylates the Smad7 lysines 64 and 70 [40], it is possible that the HDAC1 inhibition throughout isoindoline 2b offers shortly, some interesting insights to prevent the cataract formation in K14E6 mice.

Together, these results may suggest that K14E6 mice develop cataracts in a TGF-*β*-dependent manner. As TGF-*β* pathway is crucial for EMT and PCO, we propose that K14E6 mice could be an additional model for the study of TGF-dependent cataractous lesions.

## 5. Conclusion

The HPV16-E6 oncoprotein induces EMT in transgenic mice cataracts, which is a signature of PCO. The molecular mechanism may involve the TGF-*β* and Wnt/*β*-catenin pathways, suggesting that the K14E6 transgenic mouse could be a useful model for the study or treatment of EMT-induced cataracts.

## Figures and Tables

**Table 1:** Sequence of primers used for genotyping, RT-PCR, and RT-qPCR_ _^*∗*^  procedures.

Gene symbol	Forward	Reverse
E6/E7 (gene)	TTTTATGCACCAAAAGAGAACT	TACCTGCAGGATCAGCCATG
E6/E7 (mRNA)	TTTTATGCACCAAAAGAGAACT	CAGCATATGGATTCCCATCTC
p21/WAF1	TTCAGAGCCCAGGCACCATG	GGGACCCAGGGCTCAGGTAGA
COL1A1	ATGTTCAGCTTTGTGGACCTC	AGTTTGAAGCACAGCACTCG
FN1	TGCCTCGGGAATGGAAAG	ATGGTAGGTCTTCCCATCGCTATA
MMP2	TCAAGGACCGGTTTATTTGGC	GCGTCAATCTTTTCTGGGAGC
MMP9	TCCAGCGTGCCGGAAG	CCACGACCATACAGATACTGGATG
Wnt1	CAGGGTTCATAGCGATCCAT	CAAAGAGGGAGGGAGGTAGG
Wnt4	CTGGAGAAGTGTGGCTGTGA	GGACGTCCACAAAGGACTGT
Wnt5a	CTGGCAGGACTTTCTCAAGG	GTCTCTCGGCTGCCTATTTG
MTA1	AGCCCAACCCAAACCAG	GGCAATGCGTGTCAACT
E-cadherin_ _^*∗∗*^	CAGTTCCGAGGTCTACACCTT	TGAATCGGGAGTCTTCCGAAAA
GAPDH	GGTGAAGGTCGG TGTGAACG	CTCGCTCCTGGAAGATGGTG

^*∗*^Amplification conditions for PCR, RT-PCR, and RT-qPCR were 95°C, 30 sec; 60°C, 60 sec; and 72°C, 30 sec; 40 cycles.

^*∗∗*^Amplification conditions for in situ RT-PCR were 90°C, 30 sec; 60°C, 30 sec; and 72°C, 30 sec; 20 cycles.
